# Кокаин-амфетамин-регулируемый транскрипт – многообещающий омиксный прорыв в эндокринологии

**DOI:** 10.14341/probl12872

**Published:** 2022-04-30

**Authors:** Р. К. Михеев, Т. И. Романцова, Е. А. Трошина, О. Р. Григорян, Е. Н. Андреева, Е. В. Шереметьева, Ю. С. Абсатарова, Н. Г. Мокрышева

**Affiliations:** Национальный медицинский исследовательский центр эндокринологии; Первый московский государственный медицинский университет им. И.М. Сеченова (Сеченовский университет); Национальный медицинский исследовательский центр эндокринологии; Национальный медицинский исследовательский центр эндокринологии; Национальный медицинский исследовательский центр эндокринологии; Национальный медицинский исследовательский центр эндокринологии; Национальный медицинский исследовательский центр эндокринологии; Национальный медицинский исследовательский центр эндокринологии

**Keywords:** кокаин-амфетамин-регулируемый транскрипт, гипоталамус, секвенирование, иммуноферментный анализ, ожирение, метаболизм

## Abstract

**ОБОСНОВАНИЕ:**

ОБОСНОВАНИЕ. Со дня открытия кокаин-амфетамин-регулируемого транскрипта (КАРТ) прошло более четверти века. При этом данный транскрипт незаслуженно остается «в тени», несмотря на проявляемый в последние годы неподдельный интерес со стороны ученого сообщества.

**ЦЕЛЬ:**

ЦЕЛЬ. Оценить «исторические вехи» и перспективы изучения КАРТ в медицине.

**МАТЕРИАЛЫ И МЕТОДЫ:**

МАТЕРИАЛЫ И МЕТОДЫ. Поиск литературы проводили в отечественных (eLibrary, CyberLeninka.ru) и международных (PubMed, Cochrane Library) базах данных на русском и английском языках. Приоритетным являлся свободный доступ к полному тексту статей. Выбор источников был приоритетен периодом с 2019 по 2021 гг. Однако с учетом недостаточной изученности выбранной темы выбор источников датировался с 1989 г. Работа выполнена в рамках Государственного задания «Центральные и периферические патофизиологические механизмы развития болезней жировой ткани с учетом клинических и гормональных характеристик», 2020–2022 гг.

**РЕЗУЛЬТАТЫ И ВЫВОДЫ:**

РЕЗУЛЬТАТЫ И ВЫВОДЫ. Для того чтобы здравоохранение научилось более эффективно «держать удар» перед неуклонно растущей распространенностью метаболических заболеваний (ожирение, сахарный диабет и т.п.) в мире, жизненно важно открывать и изучать новые геномные, протеомные и метаболомные паттерны. В частности, одним из таких паттернов могут стать исследования, посвященные эффективности изучения возможности применения КАРТ для лечения метаболических заболеваний с позиций доказательной медицины. Чем раньше произойдет консолидация усилий клинических врачей, экспертов в области фундаментальной науки, в том числе для изучения молекулярных механизмов функционирования гипоталамо-гипофизарной системы, тем быстрее приблизится долгожданная смена парадигмы клинико-синдромальной медицины на парадигму трансляционно-персонализированной.

## Abstract (en)

**BACKGROUND:**

BACKGROUND: The cocaine-amphetamine regulated transcript has been discovered long time ago (circa over 25 years ago) but still stays not enough investigated. Just during last five years scientist’s society started providing interest to the genomic, proteomic and metabolic essence of the cocaine-amphetamine regulated transcript.

**AIM:**

AIM: The evaluation of historical pathway and perspectives of the cocaine-amphetamine regulated transcript medical investigations.

**MATERIALS AND METHODS:**

MATERIALS AND METHODS: The literature search has been provided via Russian (eLibrary, CyberLeninka.ru) and international (PubMed, Cochrane Library) databases and among articles on Russian and English languages. The main criteria for article selection was free access and 2019–2021 years of publishing. Although the introduction is based on the articles published in 1989. The present article was created according to the federal project «Central and peripheral pathophysiological mechanisms of adipose tissue diseases and their clinical and hormonal manifestations» (2020–2022)

**RESULTS AND CONCLUISON:**

RESULTS AND CONCLUISON: It is necessary to keep on investigating genomic, proteomic and metabolomic markers because they contain important clues for successful resistance against human diseases. The 21st century is the era of transformation from simple clinical medicine to personalized science. For example, researches in the area of cocaine-amphetamine regulated transcript may result in invention of genetic medicine against dangerous metabolic diseases.

75-летию академика РАН Галины Афанасьевны Мельниченко посвящается.

Исторически мировая медицина в рамках различных специальностей продолжает удивительно тернистый и одновременно благодатный путь, невзирая на смену эпох, общественно-экономических формаций, философских течений. Эндокринология, известная под определением «наука о железах внутренней секреции», прошла 4 этапа развития: 1) описательный (XVI–XVIII вв.); 2) экспериментальный (начат А.А. Бертольдом в 1849 г.); 3) выделения гормонов (1919 г. — выделение тироксина E.C. Kendall; 1921 г. — выделение инсулина F. Bunting и C. Best); 4) этап синтеза гормонов с последующим получением дериватов [1]. Открытия в области эндокринологии, спасшие и продлившие жизнь миллионам людей, были и по сей день продолжают по достоинству отмечаться Нобелевскими премиями в области физиологии и медицины [2].

Нельзя считать, что шансы новых открытий исчерпаны; напротив, внедрение большого спектра молекулярно-генетических исследований, технологий секвенирования предоставляет современным учениям огромное поле для новых научных изысканий.

Ежегодно в международных информационно-аналитических изданиях в «многотиражном режиме» публикуются тысячи гайдлайнов и протоколов исследований в рамках доказательной медицины по вопросам диабетологии, нейроэндокринологии, тиреоидологии, репродуктивной эндокринологии, что свидетельствует о заметно возрастающей роли данной специальности в повестке дня национальных (Российское общество эндокринологов (РАЭ), American Diabetes Association (ADA) и т.д.) и международных (Всемирная Организация Здравоохранения (ВОЗ)) медицинских организаций. Несмотря на экспансивный характер современной биохимии и эндокринологии, задача освоить и применить на благо человечества весь спектр гормонов остается во многом невыполненной. Авторы данной статьи представляют читателю краткие сведения об историческом пути и перспективах исследований одного из наименее изученных и наиболее загадочных нейротрансмиттеров центральной и периферической нервной системы — кокаин-амфетамин-регулируемого транскрипта (КАРТ).

Неуклонно набирающая силу пандемия метаболических заболеваний, в частности сердечно-сосудистой патологии, ожирения алиментарного генеза, сахарного диабета 2 типа, — беспрецедентно дерзкий вызов общественному здравоохранению, требующий слаженного ответа специалистов клинических и фундаментальных научных специальностей. Человечество стремглав овладевает «продуктами» научно-технического прогресса в области биоинформатики: иммуногистохимическим анализом, секвенированием генома, протеомикой и метаболомикой, генной инженерией. Новейшие технологии, несомненно, способствуют скорейшей разработке прорывных технологий в сфере лечения указанных заболеваний.

В начале 1980-х гг. американскими учеными в ходе расшифровки структуры соматостатин-подобного пептида, выделенного из гипоталамуса домашних овец, было зафиксировано появление посторонней, ранее неизвестной, аминокислотной последовательности [3]. Попытки поиска альтернативных способов лабораторного выделения данного пептида продолжались в течение следующих 14 лет. Наконец в 1995 г. в ходе эксперимента с моделированием наркотической зависимости у лабораторных мышей удалось выделить специфичный ген CARTPT (Cocaine and amphetamine-regulated transcript prepropeptide gene), что было зафиксировано в мартовском номере журнала Journal of Neuroscience (CША) [4]. Авторский коллектив вышеуказанной статьи состоял из представителей американского и австралийского научного сообщества: Oregon Health & Science University (США), Chicago Medical School (США), The University of Sydney (Австралия) Portland [3]. Таким образом, первый день весны 1995 г. ознаменовал начало новой эры в изучении гипоталамо-гипофизарного звена эндокринной системы. Описанный в статье эксперимент заключался в анализе экспрессии мРНК в ответ на введение различных психостимуляторов (кокаин, амфетамин) в организм лабораторных мышей, что привело к повышению уровня экспрессии КАРТ совместно с другим нейромедиатором — проопиомеланокортином (ПОМК) [4]. Экспрессия КАРТ была экспериментально отмечена в различных образованиях ЦНС: гипоталамусе, гиппокампе, полосатом теле. Данный феномен наблюдается не только у млекопитающих (в частности, homo sapiens), но также у рыб и птиц (в частности, у Emberiza bruniceps, известных в России как «желчные овсянки») [5–7].

Как показали более поздние исследования, проведенные индийскими учеными в 2020 г., особенностью выработки КАРТ как у рыб, так и у млекопитающих животных является циркадный сезонный ритм со склонностью к повышению в период миграции и снижению в период оседлости [7].

Период с 1995 по 2000 г. ознаменовался очередной технической революцией — открытием технологий иммуноферментного анализа (ELISA — enzyme-linked immunosorbent assay), которые навсегда вошли в повседневную медицинскую практику. Помимо решения лабораторно-диагностических задач, иммуноферментные технологии были успешно взяты на вооружение энтузиастами-патофизиологами, что позволило после серии экспериментов безошибочно найти важнейшие точки приложения эффектов КАРТ — гипоталамические ядра (дугообразное ядро, паравентрикулярное ядро, супраоптическое ядро), ядра ствола мозга (ядро одиночного тракта, латеральное парабрахиальное ядро, нижнее ядро оливы, ядро шва) и мезолимбический дофаминергический путь вентральной тегментальной области среднего мозга. По результатам ПЦР-анализа экспрессии КАРТ у лабораторных мышей была показана его лептин-зависимая посредническая роль в работе функциональной системы энергетического гомеостаза [8]; кроме того, особого внимания заслуживает глобальная роль КАРТ в формировании чувства удовольствия и положительных эмоций за успешно выполненное действие/решение в рамках системы вознаграждения мозга [9].

Вышеуказанный спектр физиологического действия КАРТ был доказан экспериментально: так, в одном из исследований сравнивались уровень выработки КАРТ и экспрессии мРНК КАРТ между стадными (контрольная группа) и изолированными (опытная группа) особями лабораторных мышей; по результатам эксперимента уровень КАРТ у социально активных мышей однозначно был выше, чем у одиночек и «аутсайдеров» в борьбе за жизненные «блага» [10]. Такое открытие может послужить многообещающим стимулом к разработке и внедрению новых групп и новых поколений уже существующих психофармакологических средств для лиц с расстройствами аутистического и дистимического спектра.

К огромному сожалению, в российском медицинском сообществе, даже на фоне эпидемиологических вызовов и активной разработки федеральных программ по борьбе с хроническими неинфекционными заболеваниями, значимость изучения КАРТ до сих пор недооценивается. В российской научной литературе имеются единичные работы, в которых упоминаются биологические эффекты КАРТ [11][12]. Определить причину низкого уровня заинтересованности российского научного сообщества данной проблемой непросто. Можно лишь предположить, что определенную роль могут играть стереотипы, основанные на сложившейся стигматизации названия. Исторический период интереса в западных странах, известных либеральным отношениям к так называемым «легким» наркотическим веществам (марихуана, гашиш), неудачно совпал с переходным периодом России в период 1990-х гг., который ознаменовался высоким уровнем активности наркотрафика из Афганистана, популярностью рэйв-культуры, злоупотреблением «клубными наркотиками», «экстази». Последствия такой стигматизации до сих пор остаются актуальными в России, несмотря на провозглашаемый открытый контур взаимодействия наших ученых с западными коллегами.

Период конца 2010-х — начала 2020-х гг. навсегда вошел в историю медицины под знаком доказательной медицины, являющейся детищем «победителя цинги» Дж. Линда (1716–1794) и А. Кохрейна (1909–1988), и успел стать наиболее продуктивным по количеству международных научных публикаций по КАРТ. Новые сведения о биологических эффектах данного нейротрансмиттера появились во многом благодаря внедрению технологий NGS-секвенирования и генной инженерии. В частности, революционные исследования по секвенированию генома наглядно продемонстрировали роль КАРТ как ключевого нейромедиатора, реализующего взаимодействие между кишечной микробиотой и структурами ЦНС посредством нейронов блуждающего нерва — активного участника регуляции пищевого поведения [13].

В феврале 2020 г. в журнале Cell Report была опубликована статья, которая позволила более пристально оценить физиологические аспекты КАРТ как важнейшего нейротрансмиттера в регуляции чувства насыщения у млекопитающих, в частности у лабораторных мышей. Экспериментальным путем было показано, что степень экспрессии КАРТ прямо пропорциональна объему потребляемой организмом пищи у живых организмов. Было выявлено, что у группы лабораторных мышей, которым КАРТ инъекционно вводили извне в проекцию ядра одиночного пути (nucleus tractus solitarius, важного центра блуждающего нерва), значительно снижался объем потребляемой пищи. Обратный, орексигенный эффект наблюдался при деструкции нейронов вышеназванного нерва или при выключении гена CARTPT. Результаты этих исследований являются надежной научной базой для разработки генно-инженерных препаратов лечения пациентов с ожирением в недалеком будущем [14].

Как известно, важнейшим гормоном в регуляции углеводного обмена является инсулин, препараты которого вводятся в организм периферическим путем при терапии сахарного диабета. Введение инсулина на периферии сопровождается не только гипогликемическим, но и анаболическим эффектом, — т.е. набором массы тела. В 1981 г. ученым удалось экспериментальным путем опробовать иной путь введения инсулина — центральный (внутримозговой). В данном случае инсулин, вводимый интравентрикулярно в желудочки головного мозга обезьянам-бабуинам, снижал объем потребляемой животными пищи. В 2021 г. данный эксперимент был выполнен с задействованием других мозговых структур лабораторных мышей (дугообразное ядро гипоталамуса), что сопровождалось подавлением выработки агути-подобного белка и нейропептида Y, ответственных за увеличение объема жировой ткани. Одномоментно с этим инсулин повышал экспрессию КАРТ и ПОМК, что приводило к снижению количества потребляемой пищи и массы тела [15][16].

Наряду с анорексигенным эффектом введение КАРТ лабораторным мышам in vivo продемонстрировало еще и нейропротективное действие данного нейромедиатора, что было подтверждено результатами электронной микроскопии биоптатов головного мозга. У особей экспериментальной группы, подвергшихся гипоксически-гипогликемическому нейрональному повреждению, после введения КАРТ было выявлено статистически значимое увеличение количества пресинаптических везикул, утолщение постсинаптического уплотнения, повышение уровня экспрессии цАМФ-зависимого транскрипционного фактора (CREB) и даже снижение экспрессии факторов образования β-амилоида. Внешне подобные артефакты проявлялись более быстрым периодом восстановления двигательной активности у мышей, которым после повреждения нервных структур вводился КАРТ. В настоящее время изучается возможность использования КАРТ с лечебно-реабилитационной целью (восстановление и реабилитация после острого нарушения мозгового кровообращения, черепно-мозговых травм и др.) [17–19]. Учитывая современные тенденции к распространению неинфекционных (в частности, сердечно-сосудистых) заболеваний, необходимо рассмотреть вопрос эффективности применения КАРТ в качестве адъювантной терапии пациентов с высоким и очень высоким сердечно-сосудистым риском на фоне ожирения и дислипидемии.

Одной из важнейших вех в короткой и насыщенной истории изучения КАРТ стало открытие в 2020 г. его собственного специфического рецептора — рецептора, сопряженного с G-белком 160 (GPR160). Экспериментальным путем на примере лабораторных животных отряда грызунов доказана способность КАРТ в качестве лиганда соединяться с рецепторами GPR160 таламуса и гипоталамуса, оказывая многовекторное действие на центральную нервную систему, а именно — модуляцию ноцицепции (передачу болевого сигнала по афферентным путям), подавление аппетита и жажды. Результаты этих исследований являются чрезвычайно перспективными с точки зрения разработки новых фармакологических агентов для лечения различных патологий, например синдрома хронической боли, ожирения и т.д. [20].

## ЗАКЛЮЧЕНИЕ

Несмотря на те обстоятельства, что со дня открытия КАРТ прошло более четверти века и он незаслуженно еще остается «в тени», его звездный час — вопрос ближайшего времени. Развитие так называемых омиксных технологий (совокупность геномных, протеомных и метаболомных паттернов) позволит за короткие сроки наверстать упущенное для решения кардинальных проблем в сфере общественного здоровья и фундаментальной науки.

## ДОПОЛНИТЕЛЬНАЯ ИНФОРМАЦИЯ

Участие авторов. Михеев Р.К. — разработка концепции, сбор и обработка материала, написание рукописи; Романцова Т.А. — идея, концепция, редакция статьи; Трошина Е.А. — идея, концепция, редакция статьи; Григорян О.Р. — редакция, обработка материала, формирование выводов; Андреева Е.Н. — редакция, обработка материала, формирование выводов; Шереметьева Е.В. — редакция, обработка материала, формирование выводов; Абсатарова Ю.С. — редакция, обработка материала, формирование выводов; Мокрышева Н.Г. — идея, концепция, редакция статьи. Все авторы внесли значимый вклад в проведение исследования и подготовку статьи, прочли и одобрили финальную версию статьи перед публикацией, выразили согласие нести ответственность за все аспекты работы, подразумевающую надлежащее изучение и решение вопросов, связанных с точностью или добросовестностью любой части работы.
